# Morphometric analysis of astrocytes in brainstem respiratory regions

**DOI:** 10.1002/cne.24472

**Published:** 2018-08-22

**Authors:** Shahriar SheikhBahaei, Brian Morris, Jared Collina, Sommer Anjum, Sami Znati, Julio Gamarra, Ruli Zhang, Alexander V. Gourine, Jeffrey C. Smith

**Affiliations:** ^1^ Cellular and Systems Neurobiology Section National Institute of Neurological Disorders and Stroke (NINDS) National Institutes of Health (NIH) Bethesda Maryland; ^2^ Centre for Cardiovascular and Metabolic Neuroscience, Department of Neuroscience, Physiology, and Pharmacology University College London London UK

**Keywords:** astrocytes, brainstem, GFAP, glia, morphology, preBötzinger complex, RRID: AB_2079751, RRID: AB_296613, RRID: AB_10013382, RRID: AB_11213580, RRID: AB_10000240, RRID: SCR_001775

## Abstract

Astrocytes, the most abundant and structurally complex glial cells of the central nervous system, are proposed to play an important role in modulating the activities of neuronal networks, including respiratory rhythm‐generating circuits of the preBötzinger complex (preBötC) located in the ventrolateral medulla of the brainstem. However, structural properties of astrocytes residing within different brainstem regions are unknown. In this study astrocytes in the preBötC, an intermediate reticular formation (IRF) region with respiratory‐related function, and a region of the nucleus tractus solitarius (NTS) in adult rats were reconstructed and their morphological features were compared. Detailed morphological analysis revealed that preBötC astrocytes are structurally more complex than those residing within the functionally distinct neighboring IRF region, or the NTS, located at the dorsal aspect of the medulla oblongata. Structural analyses of the brainstem microvasculature indicated no significant regional differences in vascular properties. We hypothesize that high morphological complexity of preBötC astrocytes reflects their functional role in providing structural/metabolic support and modulation of the key neuronal circuits essential for breathing, as well as constraints imposed by arrangements of associated neurons and/or other local structural features of the brainstem parenchyma.

## 1 INTRODUCTION

Astrocytes, the most abundant type of glial cells in the brain, control the ionic and metabolic environment of the neurons, mediate neurovascular coupling, and supply neurons with a renewable source of transmitters (Gordon, Choi, Rungta, Ellis‐Davies, & MacVicar, 2008; Philip Haydon & Carmignoto, 2006; Iadecola & Nedergaard, 2007; Magistretti, 2006; Marina et al., 2018; Mishra et al., 2016). In the rodent hippocampus and cortex, astrocytes residing in the gray matter display high anatomical complexities (Lee & MacLean, 2015; Nedergaard, Ransom, & Goldman, 2003). A single astrocyte may enwrap thousands of individual synapses (Fields, Woo, & Basser, 2015; Halassa, Fellin, & Haydon, 2009; Halassa, Fellin, Takano, Dong, & Haydon, 2007) and a multitude of parenchymal blood vessels (Iadecola & Nedergaard, 2007), putting this cell in a key position to simultaneously modulate synaptic activity as well as regulate local blood flow to match brain blood flow to the levels of neuronal activity. Although the functional significance of these complex astrocytic arrangements has not been definitively established (Bushong, Martone, Jones, & Ellisman, 2002), it has been suggested that astrocytic morphology is closely related to their crucial role in brain function (Nedergaard et al., 2003; Oberheim, Goldman, & Nedergaard, 2012; Zhang & Barres, 2010).

While it has been proposed that astrocytes can modulate the activities of CNS circuits and modulate complex behaviors (Amiri, Bahrami, & Janahmadi, 2012; Angulo, Kozlov, Charpak, & Audinat, 2004; Burkeen, Womac, Earnest, & Zoran, 2011; Fellin et al., 2009; Haydon, 2001), evidence for such modulation has only recently emerged (Gourine et al., 2010; Sheikhbahaei et al., 2018; Yang, Qi, & Yang, 2015). In the rodent brainstem, astrocytes have been shown to play a role in chemosensing and modulation of respiratory circuit activity (Angelova et al., 2015; Ballanyi, Panaitescu, & Ruangkittisakul, 2010; Eugenín León, Olivares, & Beltrán‐Castillo, 2016; Funk et al., 2015; Gomeza et al., 2003; Gourine et al., 2010; Grass et al., 2004; Huckstepp et al., 2010; Hülsmann, Oku, Zhang, & Richter, 2000; Kasymov et al., 2013; Marina et al., 2018; Mulkey & Wenker, 2011; Okada et al., 2012; Rajani, Zhang, Revill, & Funk, 2016; Sheikhbahaei et al., 2018; Turovsky et al., 2016; Turovsky, Karagiannis, Abdala, & Gourine, 2015), including the rhythm‐generating circuits of the preBötzinger complex (preBötC) located within the ventrolateral medullary reticular formation (Feldman, Del Negro, & Gray, 2013; Smith, Ellenberger, Ballanyi, Richter, & Feldman, 1991). Astrocytes are critically important for glutamate re‐cycling. Since glutamate‐mediated transmission is critical for the generation of the inspiratory rhythm (Feldman et al., 2013; Hayes, Wang, & Del Negro, 2012; Koizumi et al., 2016; Koshiya & Smith, 1999), preBötC astrocytes could potentially modulate the activities of respiratory rhythm generating neurons via control of glutamate re‐cycling (Hülsmann et al., 2000). In addition, preBötC astrocytes directly modulate inspiratory circuit activity through the release of gliotransmitters, particularly ATP/adenosine (Huxtable et al., 2009; Lorier et al., 2007; Rajani et al., 2017; Sheikhbahaei et al., 2018), prostaglandin E2 (Forsberg, Ringstedt, & Herlenius, 2017), and D‐serine (Beltrán‐Castillo et al., 2017). However, morphological arrangements of astrocytes that may reflect the complexity and functional significance of neuroglial interactions in respiratory regions and other brainstem areas have not been investigated. Considering the critical role of the preBötC, we hypothesized that preBötC astrocytes and neurons may have special structural arrangements. Recent comparative genomic, morphological, and physiological studies assessing regional transcriptional, structural and functional properties of astrocytes have provided clear evidence for regional functional diversity and specialization of astrocytes (Chai et al., 2017; Kasymov et al., 2013; Schnell et al., 2015; Turovsky et al., 2016). In this study, we reconstructed morphology and performed morphometric analyses of immuno‐labeled glial fibrillary acidic protein (GFAP) astrocytes residing within the preBötC region of adult Sprague‐Dawley rats. For comparison, we analyzed morphology of astrocytes in two other brainstem regions at the preBötC coronal plane: the nucleus tractus solitarius (NTS), and an intermediate reticular formation (IRF) region located dorso‐medial to the preBötC in which some of the neurons have been shown to have respiratory activity and a premotor function (Koizumi et al., 2013, 2008; Revill et al., 2015). The data obtained suggest that astrocytes in the preBötC are structurally more complex, which may reflect their functional role in providing structural/metabolic support and modulation of the key neuronal network essential for breathing. This complexity may also reflect constraints imposed by arrangements of associated neurons and/or other local structural features of the brainstem parenchyma.

## 2 MATERIALS AND METHODS

### 2.1 Animals

All experiments were performed on Sprague‐Dawley rats in accordance with the European Commission Directive 2010/63/EU (European Convention for the Protection of Vertebrate Animals used for Experimental and Other Scientific Purposes), the UK Home Office (Scientific Procedures) Act (1986), and the National Institutes of Health Guide for the Care and Use of Laboratory Animals, with project approval from the respective Institutional Animal Care and Use Committees. Animals were housed in a temperature‐controlled facility with 12h–12h light‐dark cycle (lights on at 7:00 A.M.). Tap water and regular laboratory rodent food were provided *ad libitum*.

### 2.2 Tissue processing and immunohistochemistry

**Figure 1:**
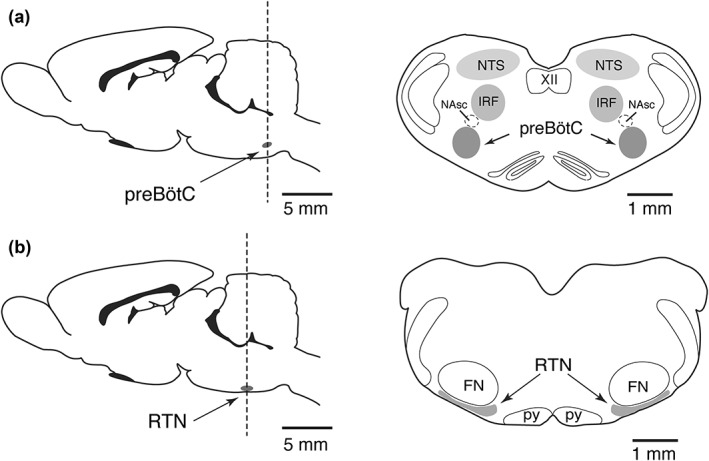
**Schematic drawings of the adult rat brain** in sagittal (*left*) and coronal (*right*) views illustrating the anatomical locations of (a) the preBötzinger complex (preBötC) region, an intermediate reticular formation region (IRF), and region of the nucleus tractus solitarius (NTS, and (b) the retrotrapezoid nucleus (RTN, gray color) in relation to motor nuclei. Other abbreviations: NAsc, semi‐compact division of nucleus ambiguus; XII, hypoglossal nucleus; py, pyramids; FN, facial motor nucleus

Five male Sprague Dawley rats (3–4 months old, ∼ 350 g) were terminally anesthetized with an overdose of urethane (3g/kg) and perfused transcardially with 250 ml phosphate‐buffered (PB, 0.1 M) solution and then with 4% paraformaldehyde (PFA) in PB solution. The brains were subsequently removed and post‐fixed in 4% PFA for 3–5 days. Brainstems were isolated and then cryoprotected at 4 °C in 30% sucrose (in 0.1 M PB‐saline solution) over 2–3 days and sectioned coronally at 40–50 µm with a freezing microtome (Leica). Floating sections (1 in 4 series) were quenched in PBS containing 10% methanol and 3% H_2_O_2_ to suppress background fluorescence. Antigen retrieval was performed in 1% citrate buffer warmed to 80 °C to unmask the proteins. Free‐floating tissue sections were incubated for 1–3 days at 4 °C with primary antibodies for GFAP (Table 1), choline acetyltransferase (ChAT; Table 1) to label motoneurons, and/or the rat endothelial cell antigen‐1 (RECA‐1; Table 1) to label vascular endothelium. The sections were subsequently incubated in specific secondary antibodies conjugated to the fluorescent probes (each 1:250; Lifescience Technologies) for 1.5 h at room temperature. Sections were mounted on slides and covered with an anti‐fading medium (Fluoro‐Gel; Electron Microscopy Sciences). Tiled images of several medullary regions of interest were obtained automatically under low magnification (10x) using an inverted confocal laser scanning microscope (Zeiss LSM 510). For morphological reconstruction and analysis of astrocytes from the selected regions, confocal image stacks of the GFAP‐positive astrocytes within the preBötC, IRF and the NTS were obtained from the same brainstem section (see Figure 1) using a high magnification, oil immersion objective (40×/1.2 NA) applying the same image acquisition settings with 1024 × 1024 pixel resolution. In order to avoid possible variations in GFAP expression during the circadian cycle (Prolo, Takahashi, & Herzog, 2005), and to minimize differences in the background fluorescence as well as in the immunostaining of astrocytes in tissue sections from different animals, all brains were fixed simultaneously using identical protocols and solutions. One investigator sectioned all the brains at the same time, and all the tissue sections were immunostained with identical solutions and processed by the same investigator.

**Table 1:** Primary Antibodies

Antigen	Description of Immunogen	Source, host species, catalog No. RRID	Dilution used
GFAP	GFAP isolated from cow spinal cord	DAKO, rabbit polyclonal, catalog #z‐0334, RRID: http://scicrunch.org/resolver/AB_10013382	1:1,000
GFAP	Purified glial filament protein	MilliporeSigma, mouse monoclonal, catalog# MAB3402C3, RRID: http://scicrunch.org/resolver/AB_11213580	1:1,000
ChAT	Whole human placental choline acetyltransferase	EMD Millipore, goat polyclonal, catalog #AB144P, RRID: http://scicrunch.org/resolver/AB_2079751	1:200
RECA‐1	Peripheral and mesenteric lymph nodes from AO rats	Abcam, mouse monoclonal, catalog #ab9774, RRID: http://scicrunch.org/resolver/AB_296613	1:1,000
GFP	Recombinant GFP protein	Chicken polyclonal, Aves Labs Cat# GFP‐1020, RRID: http://scicrunch.org/resolver/AB_10000240	1:1,000

### 2.3 Antibody characterization

The list of antibodies used in this study is given in Table 1. The goat polyclonal ChAT antibody (1:200; EMD MilliporeSigma, catalog #AB144P, RRID: http://scicrunch.org/resolver/AB_2079751) was raised against human placental choline acetyltransferase (Hersh, Coe, & Casey, 1978), and reacts with ChAT from human, pig, chicken, and rat (manufacturer's technical information). The antiserum stains a single band of 70 kDa molecular weight on Western blot (Mufson, Bothwell, Hersh, & Kordower, 1989). This goat polyclonal antibody has been used to effectively label brainstem motor nuclei (e.g., Koizumi et al., 2010, 2013, 2016; Marchenko et al., 2016). The mouse monoclonal RECA‐1 (1:1000, Abcam, catalog #ab9774, RRID: http://scicrunch.org/resolver/AB_296613) antibody was raised against peripheral and mesenteric lymph nodes from Albino Oxford rats. According to the manufacturer's technical information, this antibody reacts with vascular endothelium in rats (Hanbury et al., 2002; Loy et al., 2002; Palmer, Willhoite, & Gage, 2000). The rabbit polyclonal anti‐GFAP antibody (1:1000; DAKO, catalog #z‐0334, RRID: http://scicrunch.org/resolver/AB_10013382) was isolated from cow spinal cord and cross‐reacts with the intra‐cytoplasmic filamentous protein of an epitope of the astrocytic cytoskeleton in mouse, rat, and human [manufacturer's technical information; also see (L F Eng, Ghirnikar, & Lee, 2000)]. This antibody shows a double band (at 245–395 kDa) on Western blot (Key et al., 1993). The mouse monoclonal GFAP antibody (1:1000, MilliporeSigma, catalog# MAB3402C3, RRID: http://scicrunch.org/resolver/AB_11213580) was raised against purified glial filament protein (Debus, Weber, & Osborn, 1983) and reacts with human, porcine, chicken and rat GFAP (manufacturer's technical information). These two anti‐GFAP antibodies were found to reveal a very similar pattern of labeling in brainstem astrocytes of adult rats (see Figure 3a–c). The chicken polyclonal anti‐GFP antibody (1:1000; Aves Labs, catalog #GFP‐1020, RRID: http://scicrunch.org/resolver/AB_10000240) was obtained from animals that were immunized with recombinant GFP protein. Cells from transgenic mice expressing GFP show a single 25 kDa band in Western blot (manufacturer's datasheet).

### 2.4 3D reconstruction of astrocytes

The image stacks were imported into Neurolucida 360 (MBF Bioscience; RRID: http://scicrunch.org/resolver/SCR_001775), where reconstructions of individual astrocytes were completed with the software's tracing tools by an investigator blinded to the region of the brainstem that the images were obtained from. Astrocytes that exhibited fully intact GFAP‐immunostained processes within the sections (40–50 µm thick) containing the three regions (preBötC, IRF, NTS) were chosen for reconstruction (2–5 astrocytes per animal per region), and the cellular processes were traced throughout the entire thickness of the sections by one investigator and verified by a second investigator. The reconstruction of cellular processes was completed up to 7–11 branching orders from the soma. The finest identified and reconstructed GFAP‐immunostained branchlets were ∼0.2–0.3 µm in diameter at the highest branching order.

### 2.5 3D reconstruction of brainstem vasculature

The image stacks of regional microvasculature were imported into Neurolucida 360 (MBF Bioscience) and reconstructed using the software's tracing tools. Vessels labeled with RECA‐1 were traced throughout the entire thickness of the sections by one investigator and completeness of the tracing was verified by a second investigator.

### 2.6 Morphometric analyses of astrocytes

Cellular tracing information was then imported into the Neurolucida Explorer v. 10.42 software (MBF Bioscience), where the tracing could also be rendered as a maximum projection image (e.g., Figure 3d–f), and morphometric analyses were performed uniformly on the 3D data obtained from the selected regions. The data were used to assess several morphometric features of astrocyte, including total process length, total number of branch points, number of primary branches (processes originating from soma), and total number of terminal points. Sholl analysis (Sholl, 1953) was also performed since the complexity of astrocytes increases with radial distance from the soma. This analysis utilized regions of interest (shell volumes) between nested concentric spheres centered at the cell body (Figure 3e), with radii increasing by 5 μm, and quantified the number of branch points, number of process intersections (number of intersections between process and sphere at a given radius), and process lengths out to a given radius not including the volume of any smaller radius shells (i.e., total length of processes passing through a shell). 3D convex hull analysis, which analyses the volume enclosed by and surface area of a polygon that joins terminal points of the processes, was used to estimate the volume occupied by the astrocytic process field and surface area of the encased region occupied by an astrocyte (see Figure 3f). For normalization and comparison of cell process complexity between astrocytes from different regions, we used Complexity Index (CI), which was originally developed for the analysis of neuronal dendrites (Pillai et al., 2012) and adopted here for the analysis of astrocyte morphology. CI was defined and computed automatically from the morphometric data by the Neurolucida Explorer software (MBF Bioscience) using the following formula: (Σ terminal orders + number of terminals) × (total process length/number of primary branches), where the number of “terminal orders” for each terminal point is calculated as the number of branches that appear in proceeding backward from the defined terminal to the cell soma. Astrocyte “terminals” were defined as the smallest GFAP‐immunostained processes clearly identifiable from the last branching point in high resolution confocal images.

#### 2.6.1 Brainstem vasculature

After the microvascular 3D tracing data was imported into the Neurolucida Explorer software, the number of microvascular segments (a segment was defined as a section between two vessel branch points), total length of vessels, as well as the total volume and surface area of all the reconstructed microvessels contained within the scanned volume of each region were calculated. The volume occupied by identified blood microvasculature was normalized with respect to the total scanned volume of the region.

### 2.7 Statistical analyses

Power calculations were performed using G*POWER 3.1.93 (Faul, Erdfelder, Lang, & Buchner, 2007). The data are reported as means ± SEM, analyzed and plotted with Prism 7.0 software (GraphPad Software Inc). For statistical analyses, data were tested for normality using Shapiro‐Wilk normality test and compared by one‐way ANOVA followed by Tukey's post hoc test or Kruskal–Wallis ANOVA by ranks followed by Dunn's post hoc test, as appropriate. Differences with *p* < 0.05 were considered to be significant.

## 3 RESULTS

GFAP‐labeled astrocytes were surveyed in regions of the medulla oblongata associated with processing chemosensory information and generation of the respiratory rhythm and pattern, including preBötC, IRF, NTS and the retrotrapezoid nucleus (RTN). The anatomical locations of these regions were determined in relation to motor nuclei identified by ChAT immunoreactivity to label neurons of the nucleus ambiguus (NA), hypoglossal nucleus (XII), and facial nucleus (VII), as illustrated in Figure 1. PreBötC is located ventral to the semi‐compact division of the nucleus ambiguus (NAsc; Figure 1a). The IRF region (Koizumi et al., 2008) is located dorso‐medial to the NAsc (Figure 1a), while NTS is imaged dorsolateral to XII nucleus, within the same coronal plane containing the preBötC (Figure 1a). Images were also taken at the RTN level located ventral to the facial nucleus (FN) (Guyenet, 2014; Smith, Morrison, Ellenberger, Otto, & Feldman, 1989) (Figure 1b).

### 3.1 Morphological arrangements of brainstem astrocytes

**Figure 2:**
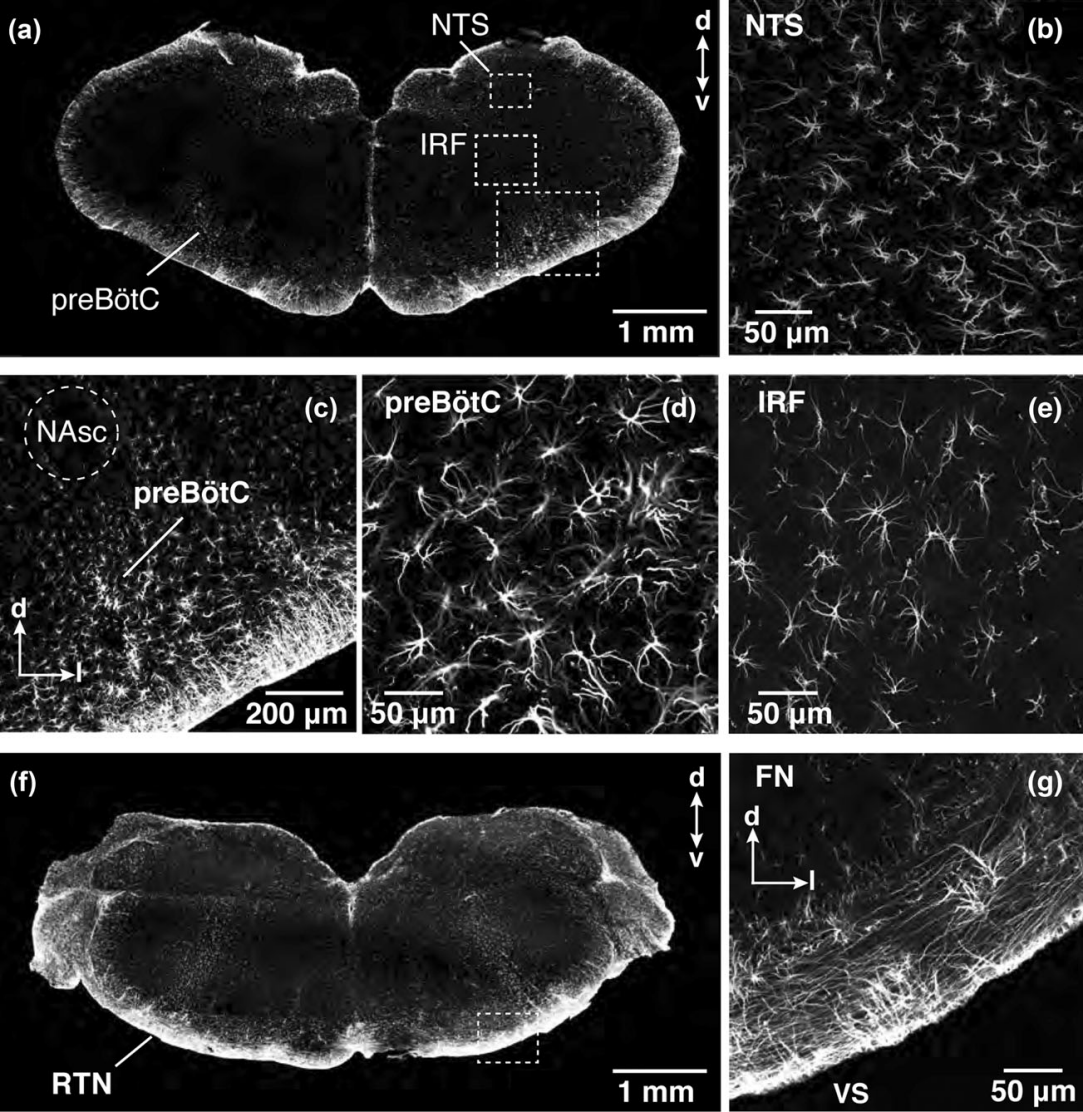
**Immunolabeled GFAP‐positive astrocytes in adult rat brainstem.** (a) Tiled low magnification confocal image of GFAP‐positive brainstem astrocytes at the medullary level of the preBötC. Dashed boxes encompass the preBötC, IRF, and the NTS regions where labeled astrocytes were imaged at high magnification and morphologically reconstructed. (b) Example of high magnification image (20 × objective) of immunostained astrocytes in the NTS. (c,d) Low (10X objective) and high (20 × objective) magnification confocal images of GFAP‐positive preBötC astrocytes. (e) GFAP‐positive astrocytes within the IRF. (f) Tiled low magnification confocal image of GFAP‐positive astrocytes at the level of the RTN. (g) High magnification image of RTN laminar astrocytes making a dense network of processes at the ventral brainstem surface. NAsc, semi‐compact division of the nucleus ambiguus; VS, ventral surface of the brainstem; d, dorsal; m, medial; l, lateral; v, ventral; FN, facial nucleus

Processes of the parenchymal astrocytes residing near the ventral medullary surface below the preBötC projected extensively into the dorsal aspect of the brainstem (Figure 2c). This organization became less apparent moving rostrally, as an extra layer of thin astrocytic processes appeared at the RTN level between the ventral surface pial membrane and the parenchyma (Figure 2f,g). Cell bodies of these relatively sparse laminar astrocytes were found to be located close to the pia mater and have numerous long processes coursing parallel to the ventral surface in the medio‐lateral plane, creating a prominent network of astrocytic fibers (Figure 2g). These GFAP‐positive processes are straighter than those of astrocytes residing within preBötC, IRF, or NTS (Figure 2g). This dense overlap of GFAP‐positive fibers was not observed in any other brainstem regions surveyed and represents a feature unique to the juxta ventral surface region of the medulla oblongata at the level of the RTN.

**Figure 3:**
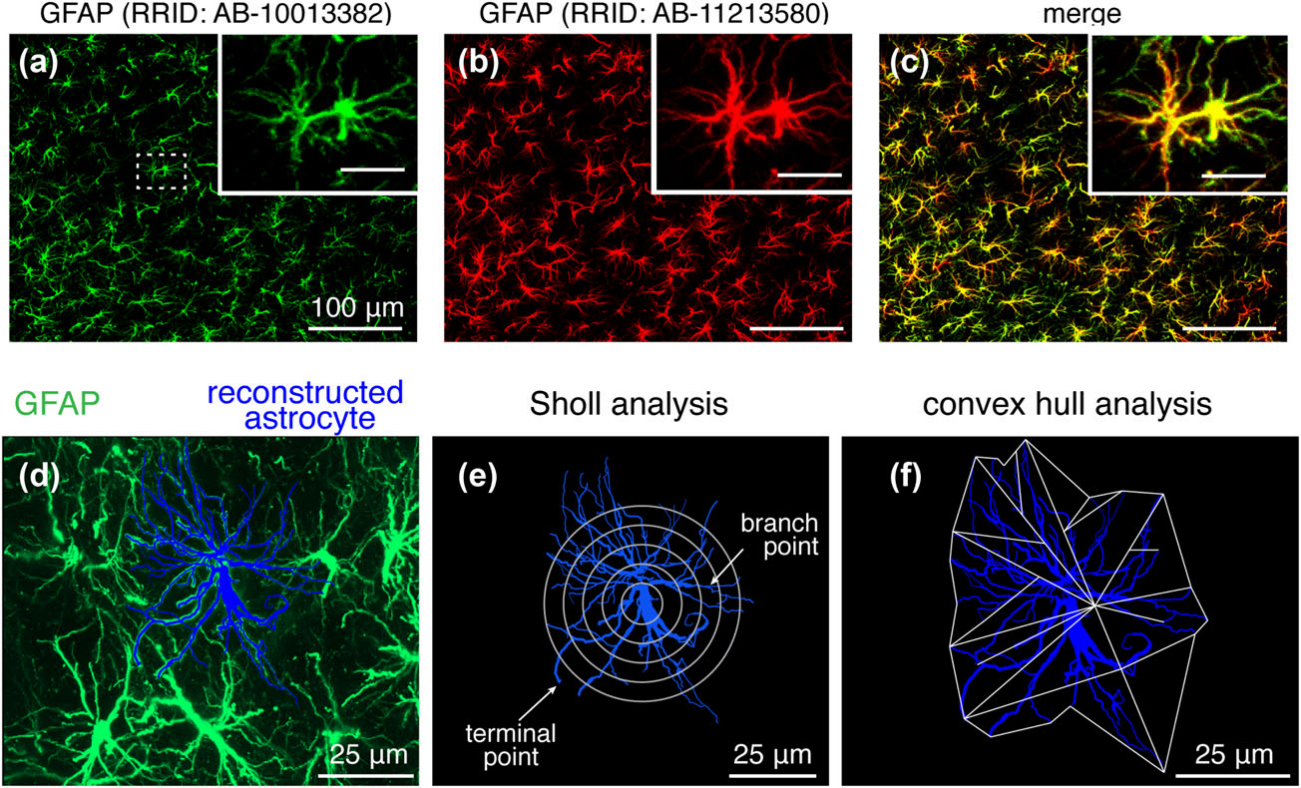
**Antibody validation and morphometric analysis of brainstem astrocytes.** (a–c) PreBötC GFAP‐positive astrocytes immunostained with rabbit anti‐GFAP polyclonal antibody (green; a) and mouse anti‐GFAP monoclonal antibody (red; b). Merged low magnification and high magnification (inset scale bar: 50 μm) images (c) illustrates colocalization of antibody labeling. (d) 2D maximum projection image of individual astrocyte (blue), morphologically reconstructed in 3D using Neurolucida 360, within the field of astrocytes identified by GFAP immunoreactivity (green; RRID: http://scicrunch.org/resolver/AB-10013382) in the preBötC region. Maximum projection image of astrocyte field and reconstructed astrocyte was rendered from Z stack of confocal images. (e) Sholl analysis to characterize astrocyte arbour complexity, including number of processes, process lengths, and number of branch points, was performed by applying nested concentric spheres increasing in size by a constant change in radius (5 μm increments) from the center of the astroglial soma (maximum projection image shown, and see Section 2.6 for detail). (f) Convex hull analysis was performed by connecting the tips of distal processes (terminal points) to generate a convex polygon (projection image shown) to determine the volume and surface area of the physical space occupied by the polygon (see Section 2.6)

In the preBötC, IRF, and NTS regions where the astrocytic processes were found to be less densely arrayed, individual astrocytes and their branched processes could be readily distinguished from patterns of GFAP immunostaining. We therefore selected IRF and NTS regions at the same medullary level with preBötC for detailed reconstruction and comparative analysis of astrocyte morphology. The densely intermingled GFAP‐stained processes of RTN astrocytes did not allow accurate tracing of processes of individual astrocytes, and, therefore, the morphology of RTN astrocytes was not assessed further.

### 3.2 Morphometric features of brainstem astrocytes

**Figure 4:**
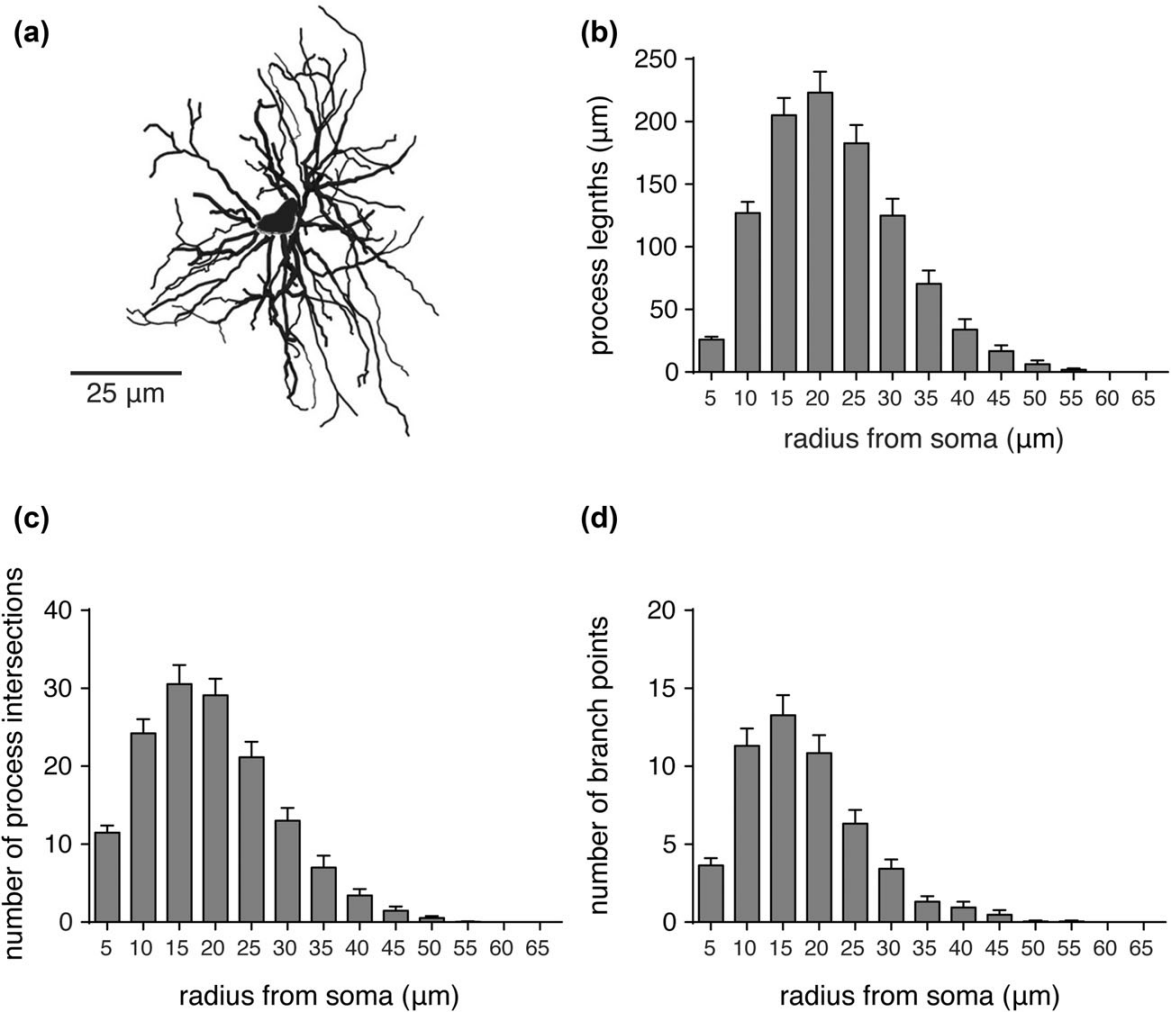
**Morphometric analyses of preBötC astrocytes**. (a) Representative maximum projection rendering from a 3D reconstruction of a preBötC astrocyte. (b–d) Summary morphometric data of process lengths, number of process intersections, and number of branch points of preBötC astrocytes (*n* = 19, from five different rats) from Sholl analysis. Radius from soma refers to the incremental spacing (5 µm) of concentric spheres centered on the cell soma. Error bars represent SEM

**Figure 5:**
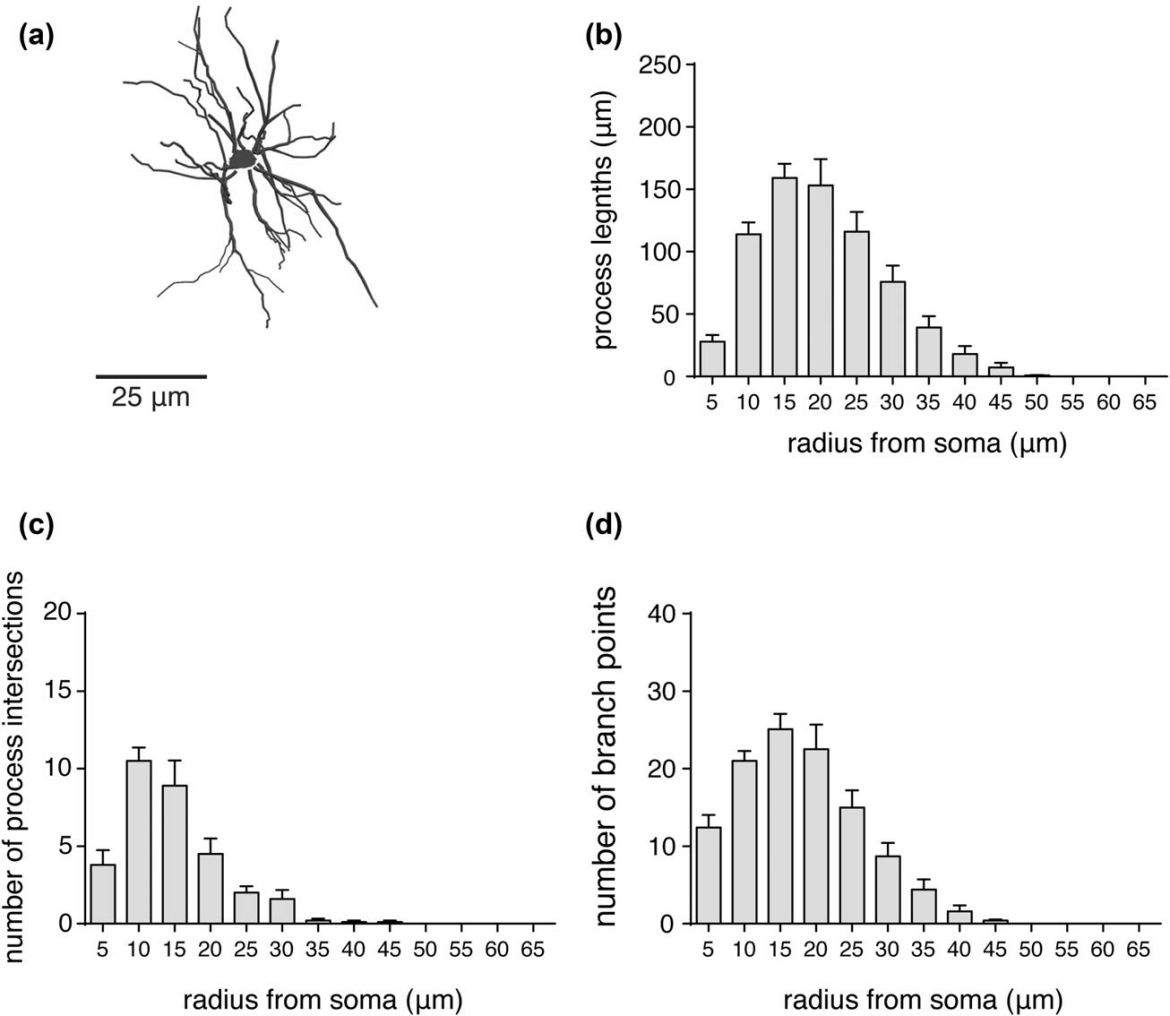
**Morphometric features of IRF astrocytes**. (a) Representative maximum projection rendering from a 3D morphological reconstruction of astrocyte in the IRF region. (b–d) Summary data from Sholl analysis (as in Figure 4) of IRF astrocytes (*n* = 10, from five different rats). Error bars represent SEM

**Figure 6:**
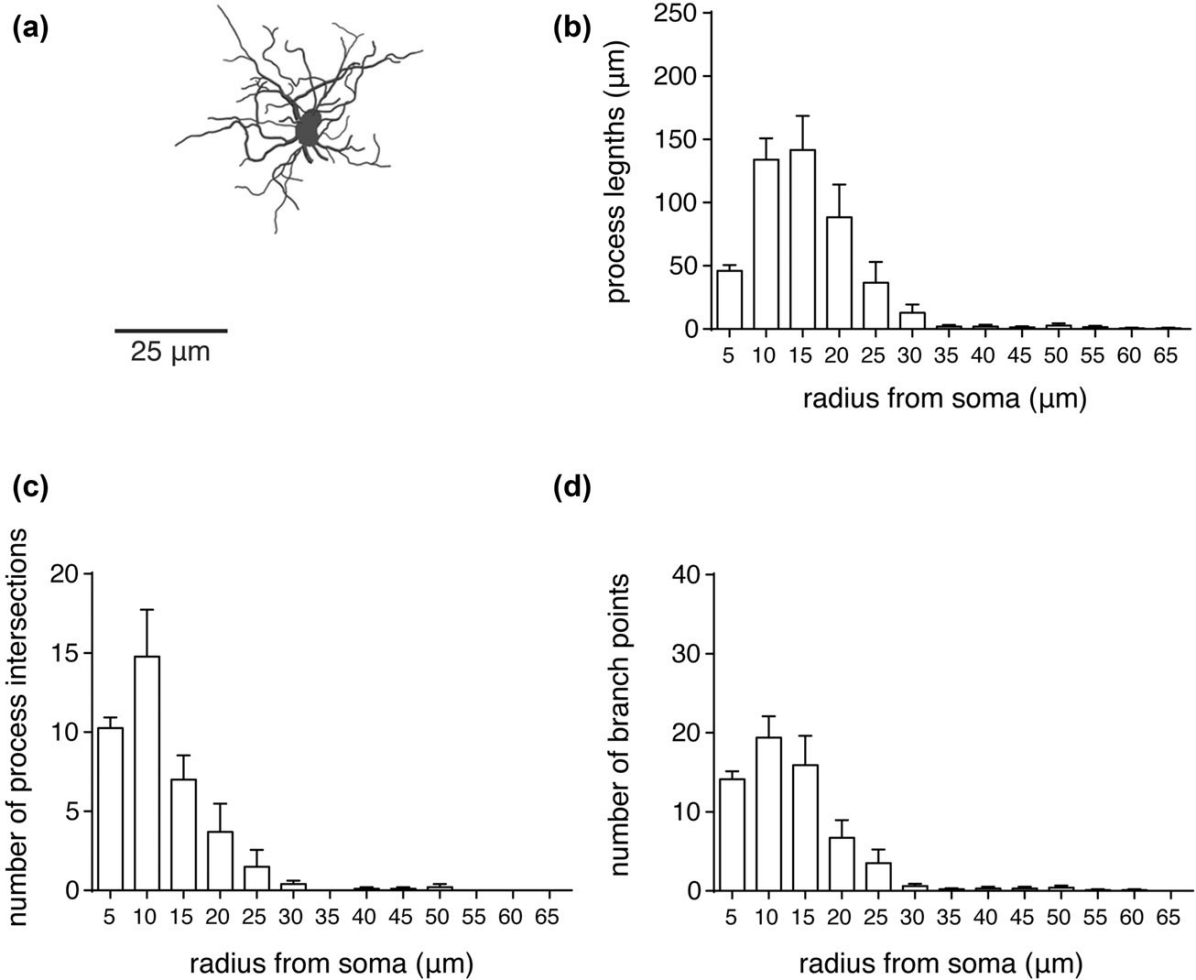
**Morphometric features of NTS astrocytes.** (a) Maximum projection rendering from a representative 3D reconstructed astrocyte in the NTS region. (b–d) Summary data from Sholl analysis (as in Figure 4) of NTS astrocytes (*n* = 10, from five different rats). Error bars represent SEM

**Figure 7:**
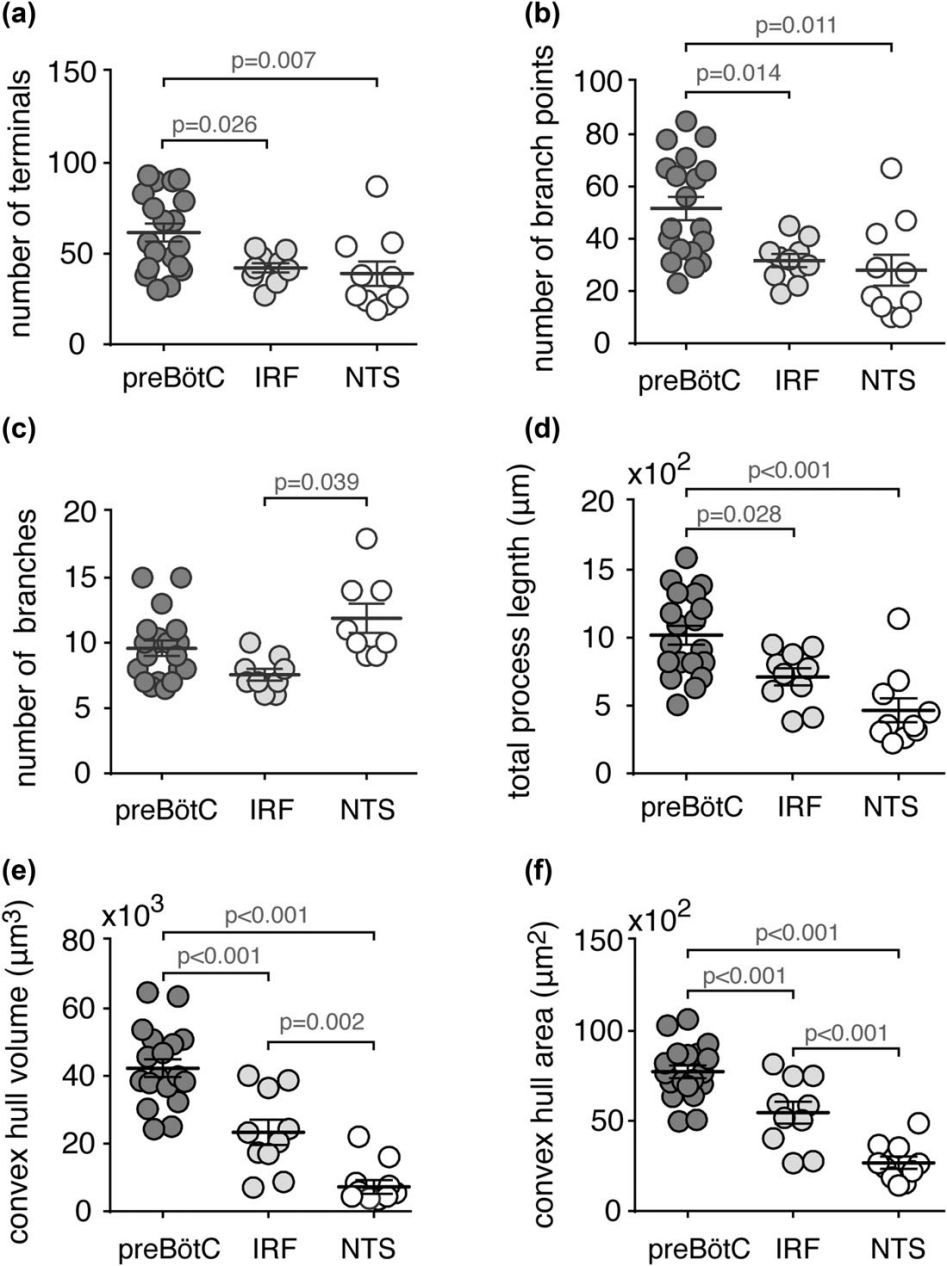
**Morphometric features of astrocytes from different brainstem regions. (**a–d) Summary morphometric data illustrating the number of terminals (a), number of branch points (b), number of primary branches originating from the soma (c), and total process length (d) of preBötC (*n* = 19), IRF (*n* = 10), and NTS (*n* = 10) astrocytes. (e,f) Summary data of the convex hull volume (e) and surface area (f) of astrocytes in the preBötC, IRF, and the NTS. PreBötC astrocytes have longer processes, more branch points and terminals, and greater convex hull volume and surface area, compared to IRF and NTS astrocytes. Data sets without *p* values indicated are not significantly different

Sholl analysis was applied to the reconstructed astrocytes from the preBötC, IRF, and NTS regions (five medullary sections in total, analyzed from 5 adult rats, see Section 2.6). The average number of branch points, number of process intersections, the total length of processes (Figures 4, 5, 6), number of process terminals (Figure 7), as well as the convex hull volume and surface area of the reconstructed astrocytes from these brainstem regions were compared (Figure 7). It was found that preBötC astrocytes have a higher average number of terminals (62 ± 5, *n* = 19 astrocytes) than IRF (41 ± 2, *n* = 10; *p* = 0.026) or NTS (37 ± 5, *n* = 10; *p* = 0.007) astrocytes (Figure 7a). The average number of branch points was higher in preBötC astrocytes (52 ± 4, *n* = 19) than in IRF (30 ± 2, *n* = 10; *p* = 0.014) or NTS (28 ± 6, *n* = 10; *p* = 0.011) astrocytes (Figure 7b). The number of primary branches (i.e., branches emanating from the soma, Figure 7c) was only different between IRF and NTS astrocytes (8 ± 0.7, *n* = 10 *vs*. 11 ± 1, *n* = 10; *p* = 0.039). The mean total length of all GFAP‐positive processes of preBötC astrocytes (1,039 ± 64 μm, *n* = 19) was significantly larger than that of IRF (691 ± 56 μm, *n* = 10; *p* = 0.028) or NTS (454 ± 80 μm, *n* = 10; *p* < 0.001) astrocytes (Figure 7d).

The convex hull volume of preBötC astrocytes (42,310 ± 2,600 μm^3^, *n* = 19) was found to be significantly larger than that of the IRF (23,351 ± 3741 μm^3^, *n* = 10; *p* < 0.001) or NTS (7,220 ± 2,056 μm^3^, *n* = 10; *p* < 0.001) astrocytes (Figure 7e). Convex hull surface area of the preBötC astrocytes (7,727 ± 345 μm^2^, *n* = 19) was also significantly larger than that of astrocytes residing in the IRF (5,461 ± 609 μm^2^, *n* = 10; *p* < 0.001) or NTS (2,676 ± 340 μm^2^, *n* = 10; *p* < 0.001) (Figure 7f). The mean convex hull volume and surface area of the IRF astrocytes were also larger than those of NTS astrocytes (*p* = 0.002 and *p* < 0.001, respectively; Figure 7f).

**Figure 8:**
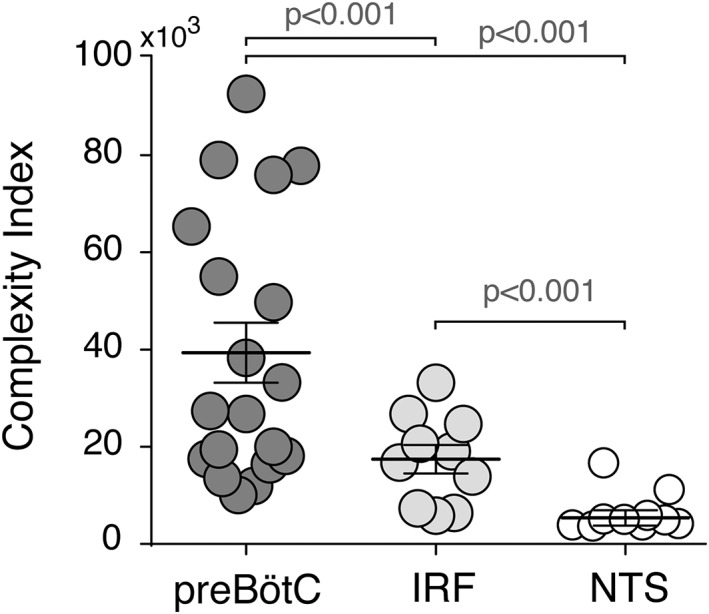
**Complexity metrics of preBötC, IRF, and NTS astrocytes.** Summary data comparing the measures of structural complexity of astrocytes obtained from the Complexity Index formula (see Section 2.6) applied to reconstructed astrocytes from the preBötC, IRF, and NTS regions. When compared to astrocytes from the other brainstem regions, preBötC astrocytes exhibit a significantly higher complexity index

To compare the overall morphological complexity of astrocytes from these brainstem regions, Complexity Index (CI) was determined using the Neurolucida Explorer software (see Section *2.6*). The CI of preBötC astrocytes (44,368 ± 5,359, *n* = 19) was found to be markedly higher than that of IRF (16,854 ± 1,545, *n* = 10; *p* < 0.001) or NTS (4,768 ± 1,168, *n* = 10; *p* < 0.001) astrocytes (Figure 8). The IRF astrocytes had a significantly higher CI than NTS astrocytes (*p* < 0.001).

### 3.3 Territories of astrocyte processes

**Figure 9:**
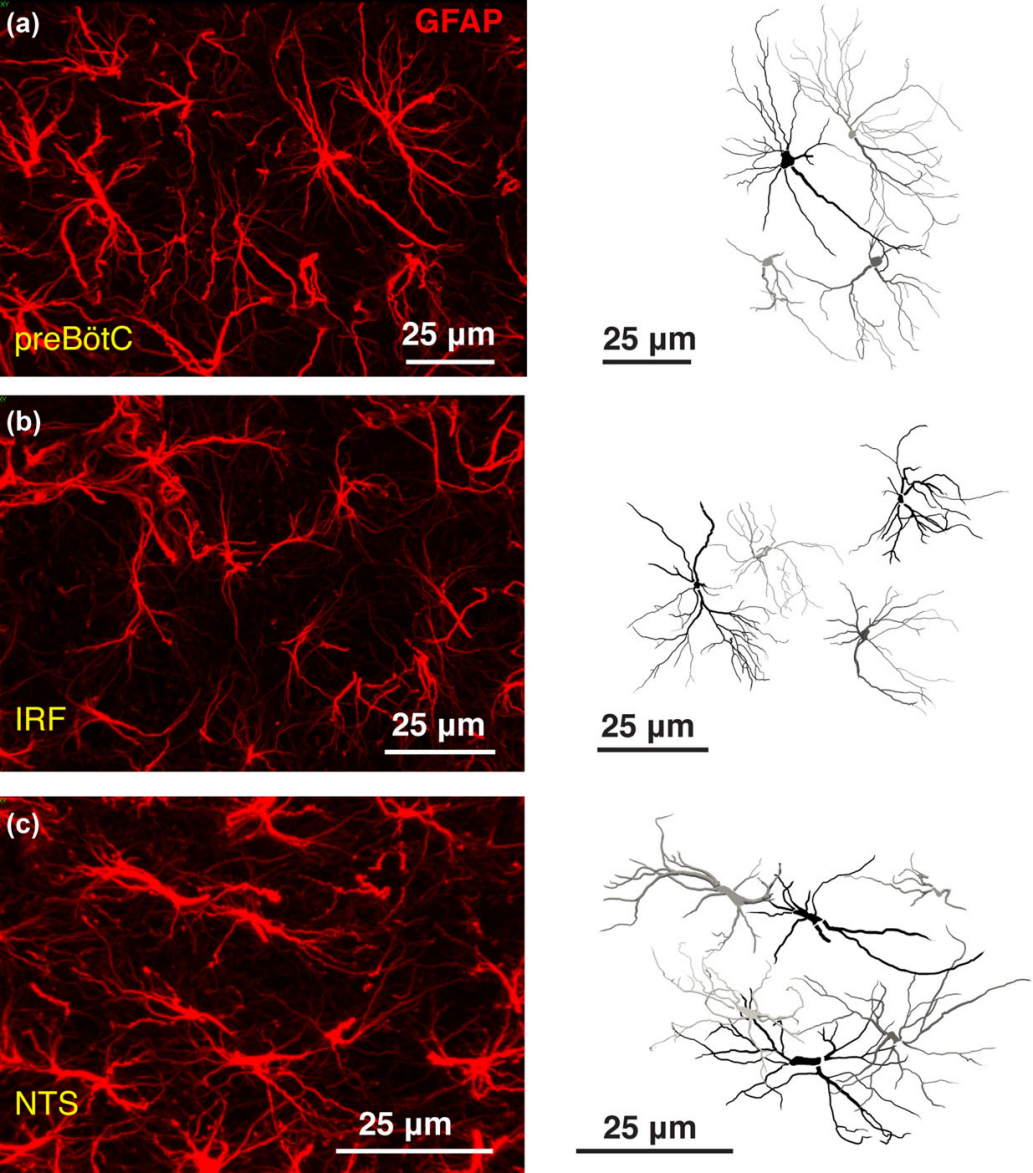
**Spatial domains of the brainstem astrocytes**. Representative confocal images of GFAP‐stained astrocytes (*left*) and full reconstruction of GFAP‐stained astrocytic process arborization (*right*) in the preBötC (a), IRF (b), and NTS (c) regions. Unlike NTS astrocytes, processes of astrocytes residing within preBötC and IRF appear to occupy distinct spatial domains

Processes of rodent protoplasmic astrocytes in the cortex, hippocampus, and striatum occupy distinct spatial domains (Bushong et al., 2002; Halassa et al., 2007; Livet et al., 2007; Oberheim et al., 2008; Ogata & Kosaka, 2002). Similarly, GFAP‐positive processes of preBötC and IRF astrocytes were found to occupy nearly exclusive territories (Figure 9). However, NTS astrocytes appear to have partially overlapping spatial domains (Figure 9), while processes of RTN astrocytes are densely intermingled (Figure 2).

### 3.4 Regional blood microvessel morphology

**Figure 10:**
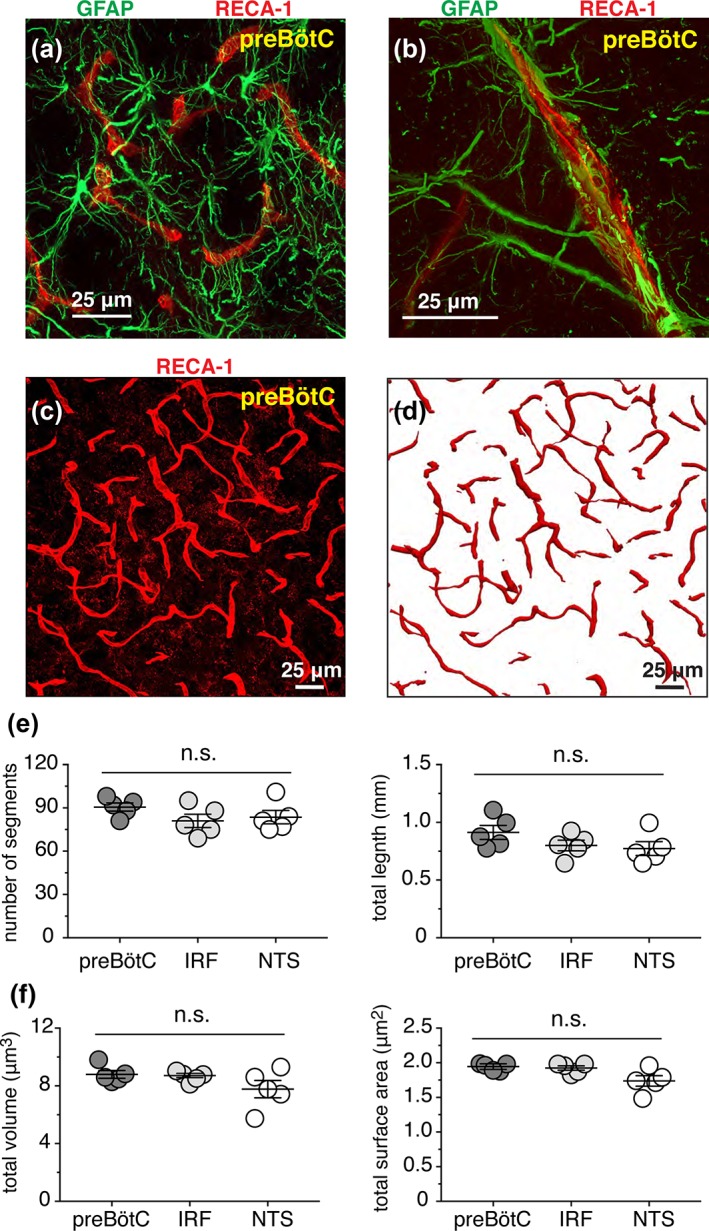
**Arrangement of blood microvessels in the preBötC**. (a,b) PreBötC astrocytes (GFAP‐immunoreactivity, green) are intermingled with the parenchymal vessels (immuno‐stained with RECA1antibody, red). (c) Representative confocal image (merged Z stack) of parenchymal blood vessels immunolabeled with RECA1 antibody in preBötC region. (d) Maximum projection image of 3D reconstructed vessels from region shown in (c). (e) Summary morphometric data illustrating the number of vascular segments (*left*) present and total length (*right*) of microvasculature in preBötC, IRF, and NTS regions analyzed. Data for each region was obtained from the same medullary section (total of five sections from five different adult rats). (f) Summary morphometric data illustrating normalized volume (*left*) and normalized surface area (*right*) of the reconstructed parenchymal microvessels in preBötC, IRF, and NTS from the same 3D reconstructions used for the analyses in (e). n.s., not significant

Brainstem astrocytes, similar to astrocytes residing within the other brain regions, make extensive contacts with all parenchymal blood vessels (Figure 10a,b). Regional differences in the morphology and complexity of astrocytes may reflect differences in the arrangements of local cerebral vasculature. Therefore, we morphometrically assessed preBötC, IRF, and NTS microvasculature within the same medullary sections (total of five sections at the same medullary level from five different animals analyzed). Figure 10c,d illustrates the 2D arrangement, represented by the maximum projection from a 3D rendered confocal image stack and reconstruction of microvessels in the preBötC region. There were no differences in the average number of blood vessel segments (91 ± 3, 81 ± 5, and 84 ± 5, *n* = 5, *p* = 0.30) and the total vascular length (1.02 ± 0.06 μm, 0.90 ± 0.05 μm, and 0.88 ± 0.06 μm, *n* = 5, *p* = 0.20), in the preBötC, IRF, and NTS regions, respectively. Moreover, the average total volume occupied by the parenchymal blood vessels was similar (*p* = 0.60) in the preBötC (8.8 ± 0.3 μm^3^, *n* = 5), IRF (8.7 ± 0.1 μm^3^, *n* = 5) and NTS (7.8 ± 0.6 μm^3^, *n* = 5) regions (Figure 10e). There were also no differences in the average total surface area of parenchymal vessels in the preBötC (1.94 ± 0.02 μm^2^, *n* = 5), IRF (1.92 ± 0.03 μm^2^, *n* = 5), and NTS (1.74 ± 0.08 μm^2^, *n* = 5) regions (*p* = 0.06) (Figure 10f).

## 4 DISCUSSION

Astrocytes, the most numerous and structurally complex glial cells of the CNS, are known to provide neuronal circuits with essential structural and metabolic support. Fine astrocytic processes are closely associated with pre‐ and post‐synaptic neurons to form tripartite synapses (Araque, Parpura, Sanzgiri, & Haydon, 1999; Halassa et al., 2009; Perea, Navarrete, & Araque, 2009; Santello, Calì, & Bezzi, 2012), potentially modulating synaptic signaling and plasticity. Astroglial signaling has been shown to modulate activities of autonomic and respiratory circuits in several regions of the brainstem containing functionally distinct neural networks including the preBötC (Forsberg et al., 2017; Sheikhbahaei et al., 2018), RTN (Erlichman & Leiter, 2010; Gourine et al., 2010; Huckstepp et al., 2010; Mulkey & Wenker, 2011; Wenker, Sobrinho, Takakura, Moreira, & Mulkey, 2012), rostral ventrolateral medulla (Marina et al., 2015, 2018) and NTS (Accorsi‐Mendonça, Zoccal, Bonagamba, & Machado, 2013; Funk et al., 2015). While it has been proposed that astrocytes may have neural circuit‐specific structural and functional properties (Chai et al., 2017), the morphological features of astrocytes residing in functionally distinct brainstem respiratory regions have not been examined. In this study, we used computer‐based 3D reconstruction of GFAP‐immunoreactive astrocytes in several brainstem regions at the medullary level of preBötC respiratory circuits of adult rats to characterize morphology of mature brainstem astrocytes.

### 4.1 Immunohistochemical labeling and reconstruction of astrocyte morphology

We employed GFAP immuno‐labeling to delineate key features of astroglial structure allowing anatomical reconstruction. GFAP belongs to the family of intermediate filament proteins that are mainly expressed in protoplasmic and specialized CNS astrocytes (Lawrence F. Eng, 1985; Jessen, Thorpe, & Mirsky, 1984). This structural protein is one of the fundamental components of the astroglial cytoskeleton and plays a critical role in the formation of complex processes of astroglia (Fuchs & Weber, 1994; Gomi, Yokoyama, & Itohara, 2010; Middeldorp & Hol, 2011; Weinstein, Shelanski, & Liem, 1991). Although GFAP immunostaining does not reveal the entire structural volume of astrocytes, this labeling approach can be used for comparative analysis of key morphometric properties of astrocytes (Eilam, Aharoni, Arnon, & Malach, 2016; Saur et al., 2014).

Other astroglial markers such as S100*β*, vimentin, glutamine synthetase, and glutamate transporters (such as GLAST or GLT) have also been used to study astroglial properties (Catalani et al., 2002). However, S100*β* and glutamine synthetase immunostaining is mainly localized in the cytoplasm of astrocytes, and only weakly label cellular processes (Wu, Zhang, & Yew, 2005). Moreover, it was reported that glutamine synthetase is expressed in oligodendrocytes and neurons (Bernstein et al., 2014; Tansey, Farooq, & Cammer, 1991). Although vimentin is also a good marker for analyzing astrocytic morphology, it is primarily expressed in developing (i.e., immature) glia cells (Dahl, Rueger, Bignami, Weber, & Osborn, 1981; Pixley & de Vellis, 1984). GLAST or GLT immunostaining is also not suitable for morphometric analysis of astrocytic processes (Saur et al., 2014), since only low quality images can be acquired (M. Zhang et al., 2011). SOX9 is another astrocyte specific marker that can be used to identify astrocytes in the adult brain (Sun et al., 2017), but SOX9 only labels the cell nucleus.

There is evidence that in hippocampal astrocytes filled with lipophilic dyes (which reveal the fine cellular processes) or immunostained with GFAP antibody (which does not delineate the finest processes), there were no significant differences between measured values of astrocyte diameter as well as the longest and thickest processes (Oberheim et al., 2008). Thus, GFAP immunostaining of astrocytes appears to be a reliable method to identify the major cellular processes of mature astrocytes. In this study, for comparative analyses of GFAP‐labeled astrocytes, the brains were fixed with the identical protocol and solutions, processed at the same time, and developed in the identical immunostaining solutions for the same period of time to standardize labeling. In addition, images used for morphological reconstruction were acquired for the different regions of interest from a single medullary section at the same level to assure standardized conditions for both immunostaining and image acquisition.

It has been estimated that GFAP‐positive processes occupy about 15% of the total volume of an astrocyte, and many of the smallest astrocytic processes (leaflets) are GFAP‐negative (Bushong et al., 2002; Oberheim et al., 2012; Ogata & Kosaka, 2002; see Supplementary Figure 1), which is a potential limitation of our approach. Thus, in order to estimate the total volume occupied by the reconstructed astrocytic processes, a 3D convex hull analysis was performed to provide a metric of the volume occupied by the astrocytic process fields, which should encase much of the field of fine processes not stained by GFAP (Supplementary Figure 1). Other approaches such as genetically‐driven expression of fluorescent proteins or injections of fluorescent dyes that have been used to label leaflets of astrocytic processes (Grosche et al., 1999; Miller & Rothstein, 2016) combined with super resolution microscopy or serial electron microscopy would ultimately be required to assess the entire structural volume of astrocytes.

### 4.2 Astroglial morphometric properties

Our data suggest that preBötC astrocytes are larger (higher convex hull volume) and structurally more complex (higher Complexity Index) than astrocytes residing within the other functionally distinct brainstem regions (IRF and NTS). Specifically, preBötC astrocytes have longer processes, more branch points and terminals, and greater convex hull volume and surface area compared to IRF or NTS astrocytes.

The data obtained also suggested that GFAP‐labeled processes of nearest neighboring astrocytes residing within the preBötC or IRF exhibit relatively little spatial overlap. However, astrocytes in the NTS and especially the RTN appear to have overlapping domains. In the rodent hippocampus and cortex, protoplasmic astrocytes residing in the gray matter occupy distinct spatial domains, with little overlap (less than 5%) (Bushong et al., 2002; Halassa et al., 2007; Livet et al., 2007; Oberheim et al., 2008; Ogata & Kosaka, 2002), though this notion has been recently challenged by the studies conducted using human (Oberheim, Wang, Goldman, & Nedergaard, 2006) and ferret (López‐Hidalgo, Hoover, & Schummers, 2016) cortical tissue. The extent of spatial overlap of astrocytic processes may have implications for neighboring astrocytes to form networks and interact functionally (Ma et al., 2016; Xu, Wang, Kimelberg, & Zhou, 2010). Structural‐functional imaging data (Chai et al., 2017) revealing the active domains is needed to confirm that preBötC and IRF astrocytes occupy nearly exclusive territories.

### 4.3 Morphometry of brainstem microvasculature

The observed differences in regional astrocytic morphological features could reflect constraints imposed by arrangements of associated neurons and/or blood vessels. We have not assessed regional somato‐dendritic morphology of neurons specifically in relation to astrocyte morphology, but we analyzed morphology of local microvasculature since it is well known that astrocytes are intimately associated with brain parenchymal blood vessels. We found, however, that the arrangement of the microvasculature in terms of average number of vessel segments, total vascular length, vascular volume and surface area in the preBötC, IFR, and NTS regions were not different.

### 4.4 Concluding remarks

Astrocytes play an important role in modulating the activity of the respiratory rhythm‐generating circuits of the preBötC (Sheikhbahaei et al., 2018). Here we show that preBötC astrocytes are structurally more complex than those residing within the functionally distinct neighboring IRF region, or the NTS located in the dorsal medulla oblongata. We hypothesize that this morphological complexity of preBötC astrocytes reflects their functional role in providing structural/metabolic support and modulation of the key neuronal network essential for breathing, and possibly also reflects constraints imposed by arrangements of associated neurons and/or other local structural features of the brainstem parenchyma.

## AUTHOR CONTRIBUTION

All authors contributed significantly to the research that led to preparation of this article. SS and JCS designed the study. SS, BM, JC, SA, SZ, JG, and RZ performed the experiments. SS, BM, and JCS analyzed and interpreted data. SS and JCS drafted the manuscript and the figures. AVG revised the article critically for important intellectual content.

## CONFLICT OF INTEREST

The authors declare no competing financial interests.
